# Antibacterial and oral tissue effectiveness of a mouthwash with a novel active system of amine + zinc lactate + fluoride

**DOI:** 10.1002/cre2.874

**Published:** 2024-07-18

**Authors:** Lyndsay M. Schaeffer, Ying Yang, Carlo Daep, Ekta Makwana, Golnaz Isapour, Norbert Huber

**Affiliations:** ^1^ Colgate‐Palmolive Technology Center Piscataway New Jersey USA; ^2^ Colgate‐Palmolive Europe Sàrl Therwil Switzerland

**Keywords:** amine, gingivitis, mouthrinse, zinc lactate

## Abstract

**Objectives:**

Reflecting the need for an effective support for the daily oral hygiene routine of patients experiencing (symptoms of) gum inflammation, a new mouthwash has been developed containing an amine + zinc lactate + fluoride system. The in vitro efficacy of this product was assessed using traditional laboratory methods, as well as novel experimentation.

**Materials and Methods:**

This mouthwash has been evaluated in a series of laboratory tests including two short interval kill tests (SIKTs), a 12‐h (longer term) biofilm regrowth assay, a plaque glycolysis assay, and an aerobic, repeated exposure biofilm model, as well as tests for soft tissue uptake and LPS neutralization.

**Results:**

Several laboratory studies demonstrate that a mouthwash containing an amine + zinc lactate + fluoride system provides short‐term and long‐term antibacterial activity. While the immediate efficacy of this formula has been shown to be driven by the presence of the amine, zinc lactate provides a long‐term antibacterial effect, as well as is able to inhibit bacterial metabolism.

**Conclusions:**

This research provides the basis for understanding the mode of action of this new mouthwash formulation and explains the previously observed clinical efficacy of this formula against plaque and gingivitis.

## 1 INTRODUCTION

Gingivitis is a site‐specific inflammation of the gums that begins with the accumulation of a plaque biofilm in the oral cavity (Chapple et al., 2018; Trombelli et al., 2018). This inflammation can be visually observed through erythema and swelling as well as the tendency for the site to bleed either spontaneously or upon mechanical probing (Löe & Silness, 1963). Gingivitis is reversible; however, if left untreated, gingivitis can develop into periodontitis (Lang et al., 2009). Mechanical removal of plaque via tooth brushing is one of the main ways that plaque accumulation can be prevented, but it is not always successful to the extent that is needed (Van der Weijden & Slot, 2015).

Recent reviews have examined the incremental benefit of chemical plaque control in the management of pre‐existing gingivitis (Figuero et al., 2020; Serrano et al., 2015). When included as part of an oral hygiene regimen, the use of a mouthwash containing antibacterial ingredients can be an effective intervention to improve plaque control and reduce gingival inflammation in the oral cavity (Sanz et al., 2020). These reviews concluded that the use of antibacterial mouthwashes as an adjunctive therapy to mechanical brushing provided significant reductions in gingivitis and plaque indices, supporting further clinical recommendations toward clinicians to consider recommending them in the context of daily oral hygiene for patients with gingivitis or periodontitis (Sanz et al., 2020; Serrano et al., 2015). It is further suggested that the use of these mouthwashes may be required, especially for those patients that are unable to effectively remove plaque by mechanical action alone (Serrano et al., 2015).

An amine and stannous fluoride (ASF) system, when delivered in mouthwashes, was shown to reduce plaque in a 4‐day plaque regrowth model (Pizzo et al., 2008), and when combined with zinc lactate (ZnL), it was shown to reduce plaque in a 3‐day regrowth model (Marchetti et al., 2017). In a 4‐week clinical study, the combination of amine and stannous fluoride in a mouthwash was shown to be more effective than a chlorhexidine mouthwash as measured by plaque index, gingival index, and gingival severity (bleeding) index (Priya & Galgali, 2015). The results from long‐term clinical studies on ASF mouthwashes have highlighted the potential of this combination as an adjunctive to brushing in patients with gum inflammation. Hoffman et al. reported a reduction in plaque index but not gingival index for ASF as compared to a water control rinse after 3 and 6 months of once daily use (Hoffmann et al., 2001). Schiffner et al. reported a reduction for ASF as compared to control in gingivitis but not in plaque after 6 months of once daily product use (Schiffner et al., 2007). Finally, Zimmerman et al. reported statistically significant reductions in both plaque index and gingivitis index in those subjects who used an ASF mouthwash once daily for 7 months as compared to those subjects who used a placebo mouthwash (Zimmermann et al., 1993). Variations in study population and design could be reasons for the observed difference in the results. These differences at baseline include the age range of the subjects (>55; 18–46; or 18–36), the number of required teeth (12 or 20), gingival conditions (a papillary bleeding index (PBI) of 1.3, and to have shown at least a 50% reduction in PBI in previous studies: chronic gingivitis with a probing depth of <5 mm or a gingival index of <1.5. All of these could impact the outcome of the results.

ZnL has previously been combined with cetylpyridinium chloride (CPC) in a mouthwash. A regimen of tooth brushing with a fluoride toothpaste followed by use of this CPC + ZnL mouthwash was shown to reduce plaque and gingivitis after 4 and 6 weeks significantly more than a regimen consisting of tooth brushing with a fluoride toothpaste and rinsing with an alcohol‐free essential oil mouthwash or than tooth brushing with a fluoride toothpaste (Langa et al., 2021). Thus, combining ZnL with an amine compound was used as a product development route, under the hypothesis that this association should provide further microbiological and clinical benefits.

Extensive clinical data show that patients suffering from plaque‐induced gingivitis may benefit from multistep oral care regimens to help control bacteria and produce measurable results, especially as tooth brushing alone often does not seem to produce the desired results. While mouthwashes are common adjuncts to tooth brushing, many products are perceived as not providing a good usage experience, altering taste and causing tooth staining concerns, (Charles et al., 2004; Ellingsen et al., 1982; Grover & Frank, 2008; Kumar et al., 2013) which can lead to less patient compliance.

To meet the needs of the patients benefiting from effective plaque control while securing an optimal adherence profile, a new mouthwash has been developed containing an amine + ZnL + fluoride system. This mouthwash has been evaluated in a series of laboratory tests including two short interval kill tests (SIKTs), a 12‐h (longer term) biofilm regrowth assay, a plaque glycolysis assay, and an aerobic biofilm model. This research provides the necessary assurance that the formula performs as expected in terms of microbiological efficacy. Separately, a 6‐month clinical study has been conducted to examine the plaque and gingival indices of this new mouthwash in comparison to a negative control mouthwash. The data reported here when combined with the clinical study will aid patients and clinicians in choosing the appropriate adjunctive interventions for supporting total oral health.

## 2 MATERIALS AND METHODS

### 2.1 Test products

- Placebo: mouthwash with no actives
- Positive control: ethyl alcohol, 100% (EtOH)
- AF: mouthwash with amine + fluoride system, 250 ppm F
- ZnL: mouthwash with ZnL, 460 ppm Zn
- AZF: mouthwash with amine + ZnL + fluoride system, 250 ppm F, 460 ppm Zn
- PBS: phosphate‐buffered saline

All mouthwashes were supplied by Colgate‐Palmolive Europe Sàrl. Ethyl alcohol was sourced from Pharmco, and PBS was sourced from VWR Life Science. The assignments of the various test products to the specific experimental procedures are described in Table 1.

**Table 1:** Distribution of test products among the various in vitro tests.

Tests	Placebo	Positive control	AF	ZnL	AZF	PBS
SIKT	X		X	X	X	X
Fluorescent SIKT	X	X	X		X	X
Plaque glycolysis	X				X	X
Aerobic biofilm inhibition	X		X	X	X	X
12 h biofilm regrowth	X				X	

Abbreviation: SIKT, short interval kill test.

### 2.2 Experimental procedures

We used several microbiological methods to assess the efficacy and potential mode of action of the mouthwash containing the amine + ZnL + fluoride system. Initial activity was quantified using a simple planktonic kill assay on whole saliva. This was followed by a short‐term exposure test on a defined consortium of oral bacteria to probe the membrane permeability of treated bacteria in a complex mix. Next, we used an in vitro plaque glycolysis assay to determine the metabolic impacts of the formula. Finally, two complex biofilm models were used to probe the effects of repeated and long‐term activity of the system on structured oral communities. Data from these in vitro studies informed our clinical design.

### 2.3 Salivary short interval kill test

Whole saliva was collected from a single, healthy adult volunteer. Saliva donors were asked to refrain from eating, drinking, and oral hygiene for a minimum of 6 h before collection, and saliva production was stimulated by chewing paraffin wax. Then, 500 μL aliquots of saliva were transferred to sterile 2 mL microcentrifuge tubes. For treatments, 500 μL of mouthwash was added and mixed completely. After 30 s incubation, the reactions were stopped by the addition of 1 mL of Dey‐Engley Neutralization Broth (D/E broth; BD Biosciences). Serial 10‐fold dilutions were made in sterile PBS (VWR Life Sciences), and 100 μL of relevant dilutions were plated on trypticase soy agar plates (TSA; BD Biosciences) containing 5% sheep's blood. Plates were incubated in a 5% CO_2_ atmosphere at 37°C for 24 h before counting. Colony forming units (CFUs) per ml from each sample were calculated. All treatments were conducted in duplicate, and the results are from a minimum of three independent experiments. Results are reported as a reduction in the log(CFUs) from an untreated sample.

### 2.4 Fluorescent short interval kill test

A five‐species consortium of bacteria was used as a model for the more complex interspecies interactions found among the 500–700 species of bacteria in the oral cavity. This is a modification of the chemostat model system described by Herles et al. (1994). Five representative oral bacteria were used: *Actinomyces viscosus*, *Lactobacillus casei* (ATCC#334), *Fusobacterium nucleatum* subsp. *polymorphum* (ATCC#10953), *Streptococcus oralis* (ATCC#35037), and *Veillonella parvula* (ATCC#17745). These five species of bacteria were grown separately in trypticase soy broth (BD Biosciences) with 2% yeast extract (TSB‐YE) for *A. viscosus*; brain heart infusion (BHI; BD Biosciences) for *L. casei*, fluid thioglycollate medium (FTG; BD Biosciences) for *F. nucleatum*; trypticase soy broth (TSB; BD Biosciences) for *S. oralis*; and Schaedler broth (Himedia Laboratories) for *V. parvula*. Individual cultures were used to inoculate a 500 mL spinner flask containing 50% McBain medium (ref). This mixed culture was maintained for a maximum of 5 days. Each day, approximately 250 mL of the culture was removed to a sterile bottle and replaced with 250 mL of sterile medium to allow the culture to keep growing. Before each use, the composition of the culture was verified via Gram staining.

To allow for the quantification of the mixed species population, a fluorescent dye‐based modification of a traditional SIKT was used. In this assay, 2 mL aliquots of the mixed species inoculum described above were transferred to sterile microcentrifuge tubes. Bacteria were pelleted by centrifugation and resuspended in 100 μL of sterile PBS. Samples were treated with 100 μL of the indicated mouthwash for a final concentration of 50%. Following 5 s of treatment, samples were treated with 1.350 mL of D/E broth. Neutralized samples were pelleted, washed in 1 mL of sterile PBS and then resuspended in 150 μL of sterile PBS. Fifty microliters aliquots were plated in triplicate in a sterile 96‐well microtiter plate. Samples were treated with 50 μL of BacLight Live Dead stain (Life Technologies) as described in the manufacturer's instructions. Samples were read in a fluorescence plate reader (EnVision; PerkinElmer) at an excitation wavelength of 485 nm and emission wavelengths of 535 and 595 nm. Viability was calculated by determining the ratio of average green (535 nm) to red (595 nm) fluorescence for the triplicate samples and presented as a percentage of viable bacteria relative to a control treated with sterile PBS alone. Results represent a minimum of four independent experiments.

### 2.5 In vitro plaque glycolysis

The in vitro plaque glycolysis assay is an adaptation of the plaque glycolysis portion of the PGRM assay devised for assessing the efficacy of antibacterial formulations (Myers et al., 2019). In this assay, saliva‐derived biofilms were grown on hydroxyapatite (HAP) discs (Himed) held in a vertical position using the lid developed for the Amsterdam Active Attachment Model (Exterkate et al., 2010). Two milliliters of whole saliva was used to inoculate 50 mL of McBain medium, and 2 mL of medium was used per disc. Plates were incubated for 48 h in a 5% CO_2_ atmosphere. The 2‐day old biofilms were treated for 2 min with the indicated mouthwash, rinsed and then transferred to a glycolysis medium consisting of 0.3% TSB and 0.5% sucrose, adjusted to pH 7.2. Biofilms were incubated for 6.5 h before the pH of each well was measured, and the results are reported as the pH change of the sample. Each treatment was done in triplicate and at least three independent experiments were performed.

### 2.6 Aerobic biofilm inhibition

The aerobic biofilm model was developed as an in vitro model that mimicked the growth conditions, microbial diversity, doses, and treatment times seen in clinical product use. In this model, biofilms were grown on hydroxyapatite discs held in a vertical position by a special steel lid initially developed for the Amsterdam Active Attachment Model (Exterkate et al., 2010). For the initial inoculum, whole saliva was harvested from a single donor. 25% whole saliva was inoculated in fresh SHI medium, and 1.5 mL were transferred to each well of a 24 well plate (Tian et al., 2010). Sterile discs were incubated in the inoculum for at least 4 h before the first treatment.

Treatments were performed in undiluted mouthwash. 1.5 mL of treatment was placed in the appropriate wells of a sterile 24 well plate. Inoculated discs were transferred to this plate and incubated for 30 s at room temperature with vigorous shaking on an orbital shaker. Following treatment, discs were rinsed for 5 min in a plate containing 1.5 mL/well of fresh, sterile 0.25x TSB with the same vigorous shaking. Discs were then transferred to a plate containing fresh SHI medium and transferred to a 37°C incubator supplemented with 5% CO_2_ and incubated overnight. For the subsequent 3 days, plates were treated two times per day at least 4 h apart.

Following treatment in the morning of the fifth day, plates were incubated for 2–3 h to allow the bacteria to recover. Discs were then transferred to a plate containing 1 mL of sterile water per well. Discs were sonicated for 2 min in 30 s pulses on each side to remove biofilm. Finally, discs were removed from the wells and the plate was sonicated one additional time for 2 min in 30 s pulses, to pellet any HAP suspended in the solution.

Biofilm bacteria were subjected to several different measurements. The absorbance at 610 nm is read as an indicator of total biomass. Bacteria were stained using the BacTiter Glo ATP assay (Promega) per the manufacturer's instructions as a measure of the metabolic activity of the biofilm. Finally, bacterial cells were stained using the BacLight Live/Dead bacterial viability kit (LifeTechnologies) following the manufacturer's instructions. All measurements were reported as a percent reduction in signal relative to an untreated control. Results represent a minimum of three independent experiments.

### 2.7 Lipopolysaccharide (LPS) neutralization

LPS was detected and quantified using Pierce LAL Chromogenic Endotoxin Quantitation Kit (Pierce Biotechnology). Mouthwashes were diluted to lower concentrations in endotoxin free water (final test concentration at 20x and 50x dilutions). 96‐well microplates were first equilibrated in a heating block for 10 min at 37°C. Fifty microliters of standard *Escherichia coli* LPS solutions (1, 0.5, 0.25, 0.1, 0 EU/mL) were dispensed into the microplate wells to generate a standard curve. Twenty‐five microliters of test solution was dispensed into each microplate well, mixed with 25 μL of 2 EU/mL either *E. coli* LPS or precalibrated *Porphyromonas gingivalis* LPS solution. Next, 50 μL of Limulus Amebocyte Lysate (LAL) reagent was added to each well. Plates were shaken gently and incubated for an additional 10 min at 37°C, followed by the addition of 100 μL of chromogenic substrate and incubation for 6 min at 37°C. Finally, 50 μL of stop reagent (acetic acid) was added, and the absorbance was measured at 405 nm on a microplate reader (Envision, PerkinElmer). Standard curves were generated based on the absorbance of the standard *E. coli* solutions. The concentrations of LPS in ingredient solutions were calculated based on the standard curve. The LPS inhibition ratio was calculated as follows:

$$\%\mathrm{LPS}\;\mathrm{inhibition}=\frac{\mathrm{Standard}\;\mathrm{LPS}\;\left(\frac{1\mathrm{EU}}{\mathrm{mL}}\right)-\mathrm{detected}\;\mathrm{LPS}}{\mathrm{Standard}\;\mathrm{LPS}\;\left(\frac{1\mathrm{EU}}{\mathrm{mL}}\right)}\times 100.$$

### 2.8 Twelve‐hour biofilm regrowth

Unstimulated saliva was collected from individuals who generally have good oral health. Saliva was collected from overnight fasted, unbrushed donors. All donors were prescreened to ensure that they are not brushing with a therapeutic toothpaste before donating saliva. Saliva‐derived biofilms were cultured vertically on HAP disks at 37°C under 5% CO_2_. The biofilms were cultured in McBain media (ref) supplemented with 5 µg/mL (final concentration) hemin and 1 µg/mL final concentration vitamin K for a total of ~60 h. The media was replaced twice daily at ~12‐h intervals. The resulting biofilm culture was treated with the selected mouthrinses for 30 s under circular agitation (80 rpm). The biofilms were washed twice with sterile deionized water (dH_2_O) by dipping the treated biofilms five times in sterile deionized water each time. Following treatment, the biofilms were allowed to recover for approximately 2 h in sterile dH_2_O at 37°C before harvesting the biofilms. Biofilms were dislodged from the HAP disks and resuspended in sterile dH_2_O by gently vortexing for 30 s. The treated bacterial suspensions were inoculated in 96‐well polystyrene plates containing BHI broth supplemented with 2% yeast extract (final concentration) to a final optical density of 0.2 (Absorbance = 610 nm) and final culture volume of 200 µL. The bacterial suspensions were cultured overnight at 37°C. The bacterial density was measured (Absorbance = 610 nm) hourly over 12 h. A total of three experiments were compiled and analyzed. Each experiment consisted of four biofilms per treatment group. Results were analyzed based on one‐way analysis of variance using Tukey's multiple comparison test.

### 2.9 Zinc deposition on MatTek EpiGingival^TM^ tissues

MatTek EpiGingival^TM^ tissues (MatTek Life Sciences) were treated with Mouthwash (1 mL/tissue) for 2 min at room temperature. Tissues were washed with PBS (2 mL) three times and transferred into fresh tubes, one tissue per tube. Tissues were digested with nitric acid (70%, 0.5 mL) at room temperature overnight. Digested samples were diluted with deionized water (4.5 mL to a total volume of 5.0 mL), followed by centrifugation of the tubes at 4000 rpm for 10 min. The supernatant of each sample was transferred into a fresh tube for analysis with inductively coupled plasma optical emission spectroscopy (ICP‐OES).

### 2.10 Zinc deposition imaging

Gingival cells were collected from healthy adult volunteers by gently swabbing the gum surface at sites with no visible signs of inflammation with a sterile cotton swab. Cells were resuspended in 1 mL of sterile PBS and centrifuged briefly to pellet the cells. Supernatants were removed and cells were resuspended in 1 mL of mouthrinse for 1 min before centrifuging and removing. Cell samples were resuspended in 1 mL of Hanks' Balanced Salt Solution (HBSS) (VWR Chemicals), and 2 uL of 2.4 mM zinquin ethyl ester (Sigma‐Aldrich) stock solution was added. Samples were incubated at 37°C for 30 min. Cells were washed in 1 mL of HBSS, resuspended, and imaged under epifluorescence microscopy.

### 2.11 Statistical analysis

All statistical analyses were performed using standard methods as noted in results and figures. Samples sizes were determined by power calculations based on previous experimental data. Significance threshold for all analysis was *p* > .05.

## 3 RESULTS

### 3.1 Short interval kill test

**Figure 1:**
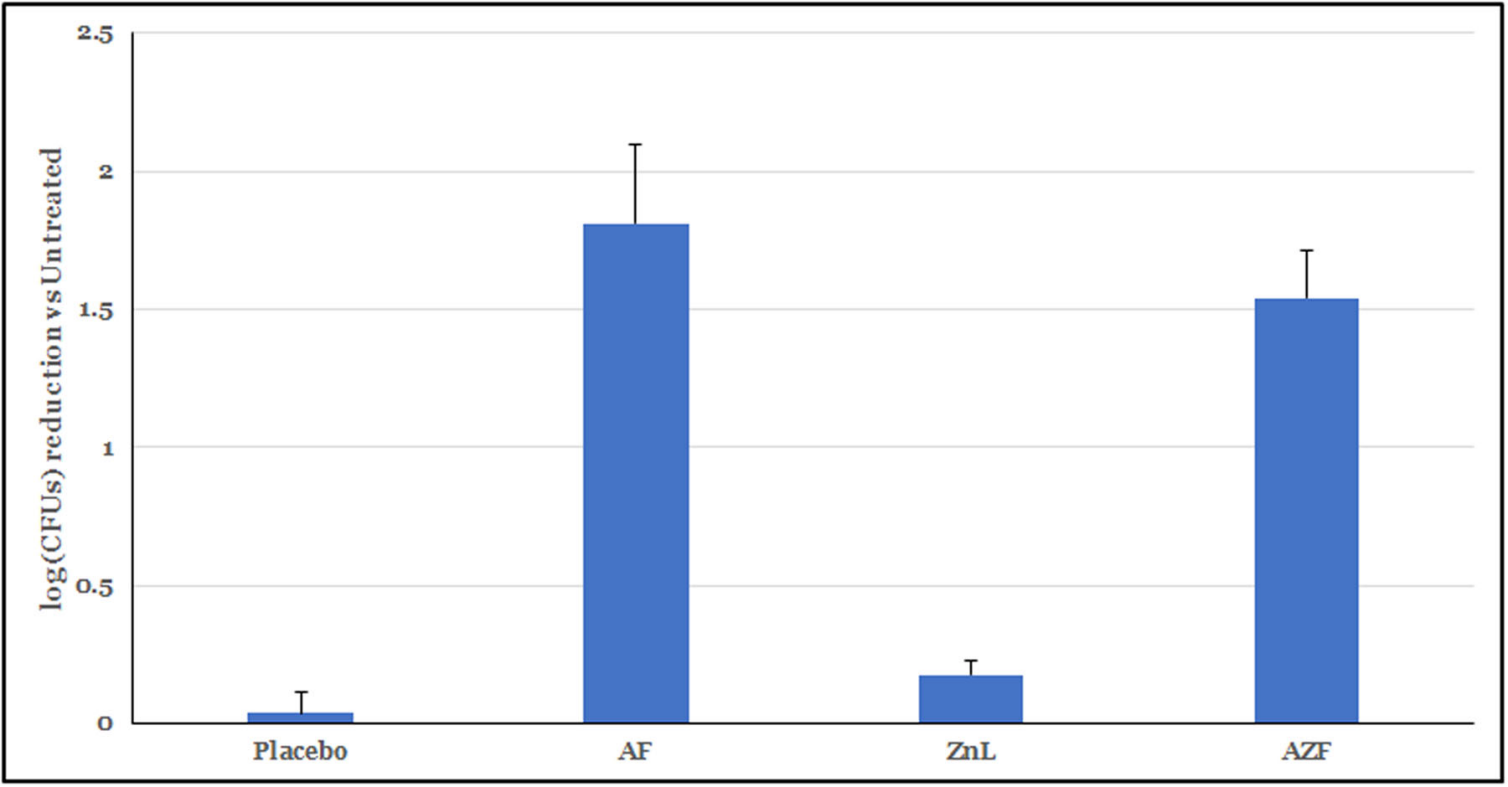
Salivary SIKT of mouthwash formulations. Following 30 s treatment, the AZF mouthwash gave a reduction in log(CFUs) that was statistically significantly different from the placebo mouthwash. The AF mouthwash and ZnL mouthwash were also statistically significantly different from the placebo mouthwash (*p* < .05 by Student's *t* test). The majority of the immediate antibacterial effect is derived from the amine base in the formula.

Planktonic SIKTs are useful methods for identifying the rapid antibacterial effects of formulations. While traditional SIKT studies focus on effects on single, laboratory‐cultured species of bacteria, this is not how bacteria exist and develop in the oral cavity. To get a clearer picture of the impact of the tested mouthrinses on oral bacteria, we used a whole saliva inoculum as a representative of the first bacterial communities that the tested formulas would encounter. This method allows for a more realistic estimation of the immediate effects of the active formulations following short exposures. As can be seen in Figure 1, the AZF mouthwash performed significantly better than a placebo mouthwash. Mouthwashes containing either AF or ZnL alone demonstrated that the majority of the short‐term antibacterial effects in the AZF mouthwash were driven by the presence of amine.

### 3.2 Fluorescent SIKT

Traditional plating‐based SIKTs are limited in their ability to quantify the effect of some active ingredients due to the general limitations of bacterial plating. The two‐component fluorescent dye system used in the BacLight viability assay looks specifically at membrane permeabilization as a measure of viability. This method relies on a two‐component system of fluorescent DNA‐intercalating dyes. The first stain is only able to penetrate bacterial cells with membrane damage, while the counterstain stains all of the cells present.

**Figure 2:**
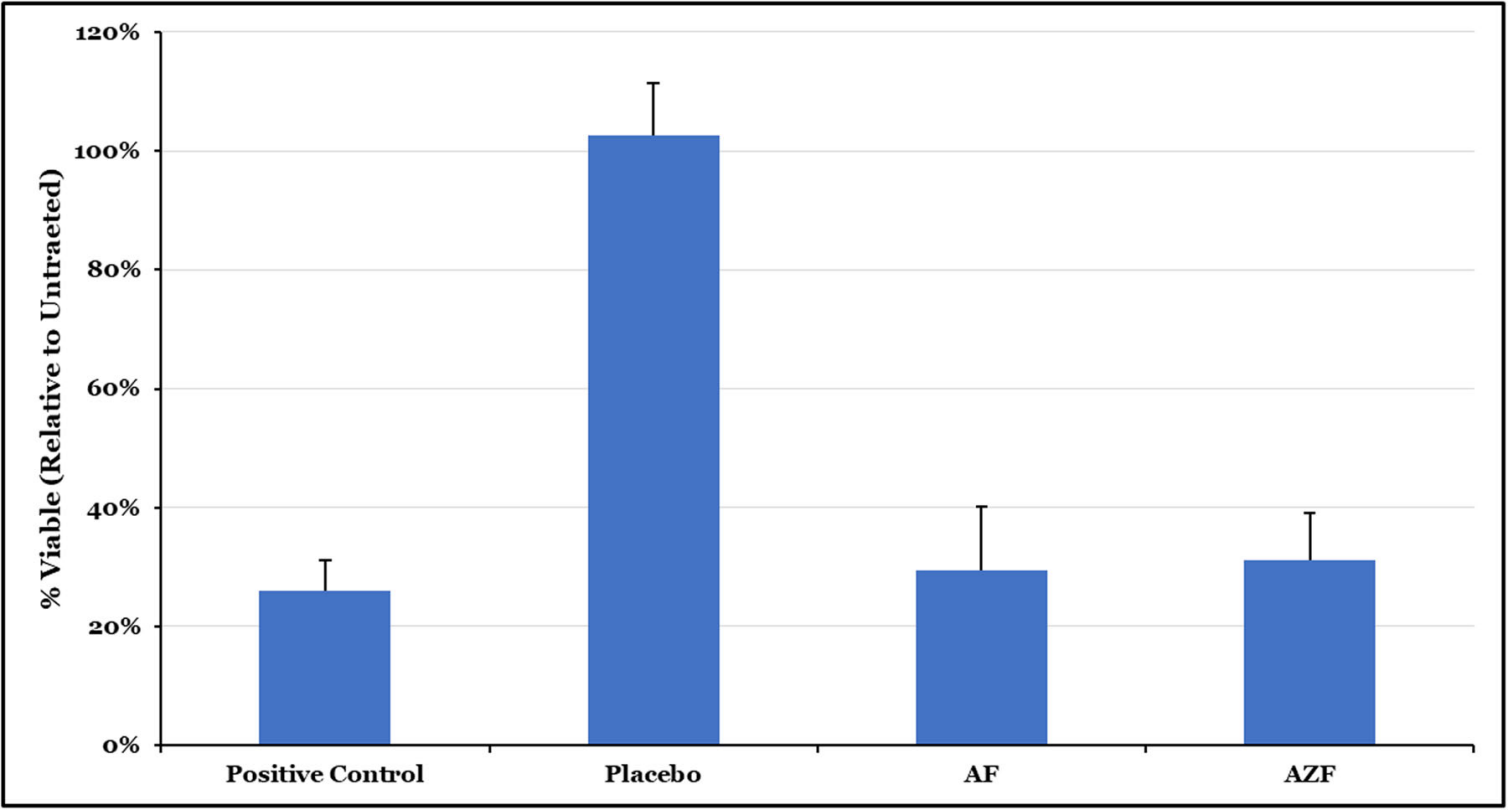
Fluorescent SIKT study. Samples that were treated for 5 s with the indicated treatment performed better than the placebo mouthwash at permeabilizing the membranes of bacterial cells. The AF mouthwash and AZF mouthwash performed similarly to the positive control (EtOH) and significantly better than placebo mouthwash (*p* < .05).

The AF mouthwash and the AZF mouthwash performed similarly to the positive control, 100% EtOH, and significantly (*p* < .05) better than the placebo mouthwash (Figure 2). Additionally, the AF mouthwash performed similarly to the AZF mouthwash in this assay. This demonstrates two things: (1) the immediate efficacy of this mouthwash is driven largely by the AF and (2) the killing of bacteria by AF occurs via a permeabilization of bacterial cell membranes.

### 3.3 In vitro plaque glycolysis

**Figure 3:**
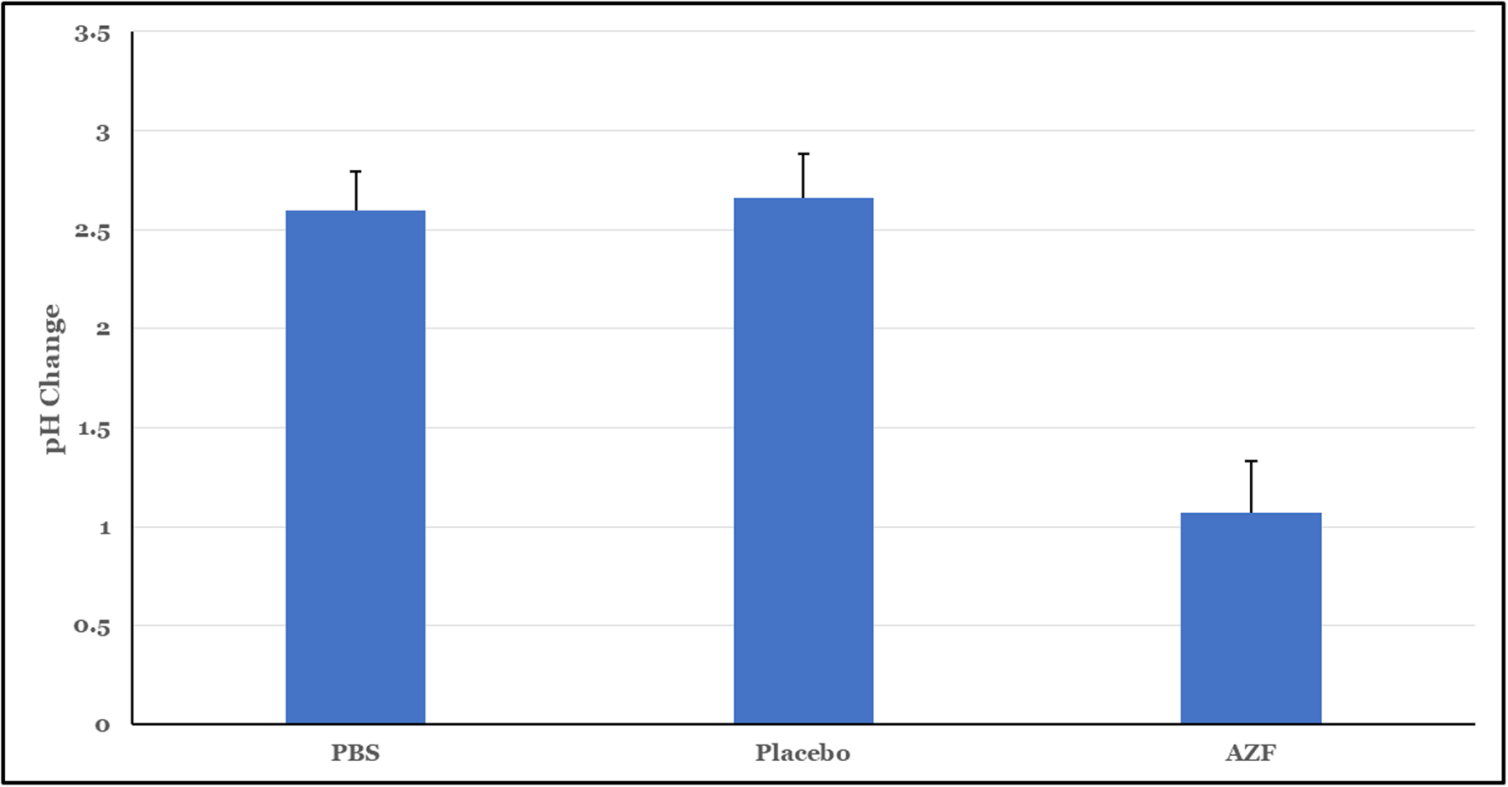
In vitro plaque glycolysis inhibition by mouthwashes. In this assay, the AZF mouthwash prevented acid production of biofilm communities when compared to PBS and placebo treatments, indicating an alteration of metabolic activity (*p* < .05).

Metabolically active oral communities are known to produce acid in response to sucrose challenge. The in vitro plaque glycolysis model was adapted from the in vivo plaque glycolysis and regrowth method that has previously been used to describe the inhibition of metabolic activity following single treatments (Kasturi et al., 1995). This model allows us to look at the short‐term effects of a formula on an oral biofilm community. Biofilm‐dwelling bacteria are less susceptible to antibacterial agents and, therefore, represent a more real‐life challenge than the simpler planktonic assays described previously. As can be seen in Figure 3, the AZF mouthwash inhibited the metabolic activity of saliva‐derived biofilms following a single treatment and was statistically significantly different (*p* < .05) from placebo and PBS by Student's *t* test.

### 3.4 Aerobic biofilm inhibition

**Figure 4:**
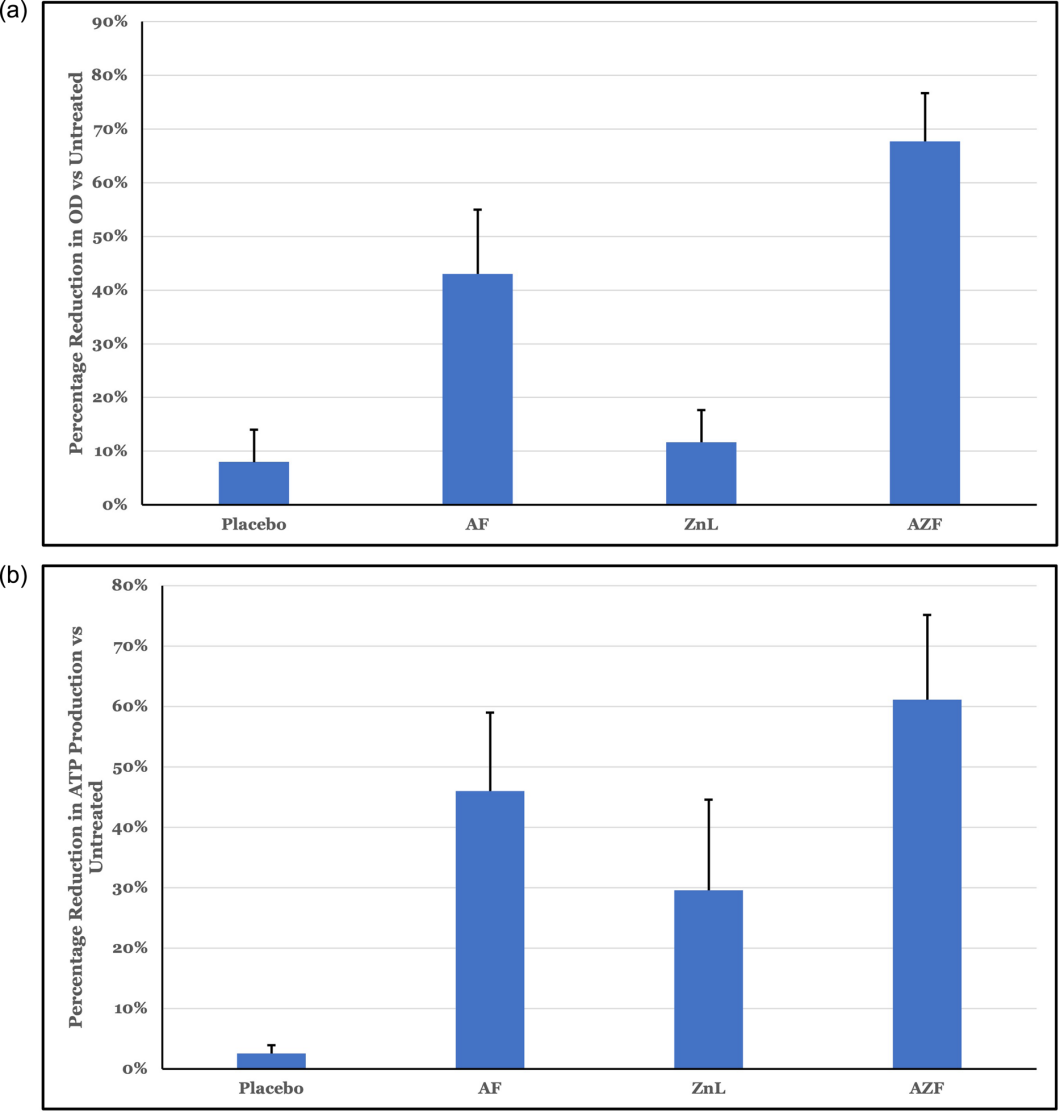
Biofilm inhibition by repeated treatment with mouthwashes. Repeated treatment with mouthwashes over a 5‐day period significantly inhibited (*p* < .05) both (a) the total biomass and (b) the ATP activity of model oral biofilms as compared to the placebo.

Biofilm models represent the closest in vitro approximation of plaque community behavior in response to treatment. To facilitate better understanding of the in vivo impact of formulas, we developed an aerobic biofilm model that mimics the behavior and resilience of plaque near the gumline. As can be seen in Figure 4, the AZF mouthwash provided significantly greater in vitro efficacy (*p* < .05) as compared to either the AF or ZnL mouthwashes. The OD_610_ of the community (Figure 4a) provides an indication of the total biomass of the resultant biofilms, while the relative ATP activity (Figure 4b) measures the overall energetic activity of that community.

### 3.5 Lipopolysaccharide neutralization

**Figure 5:**
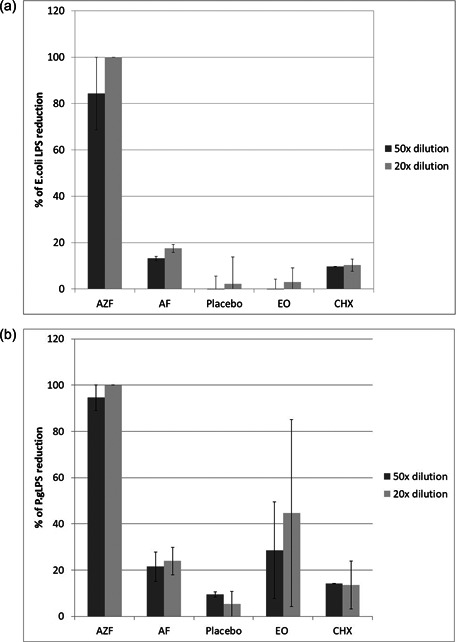
Neutralization of LPS by mouthwash treatment. Samples of (a) *Escherichia coli* or (b) *Porphyromonas gingivalis* LPS were treated with the indicated mouthwash, and samples were then measured using the Pierce LAL Chromogenic Endotoxin Quantitation kit as an endpoint measurement. Treatment with the AZF mouthwash reduced the measurable, active LPS in all cases.

In addition to providing long‐term antimicrobial activity, the addition of ZnL to this mouthwash formula provides the ability to also reduce the pathogenic activity of oral bacteria. In other settings, Zn ions are known to bind to bacterial LPS and mitigate the inflammatory response. Using an LPS quantification kit, we found that the AZF mouthwash at a 50x dilution reduced measured LPS by 84% and at a lower 20x dilution eliminated measurable *E. coli* LPS (Figure 5a). Comparatively, an AF only or a placebo mouthwash reduced LPS by less than 20%. LPS reductions from a competitive EO or CHX mouthwash had similarly minimal effects on LPS.

In a more orally relevant model using *P. gingivalis* LPS (Figure 5b), we again saw an 89% reduction in LPS at the 50x dilution and, again, a 100% reduction at the 20x dilution. Comparatively, the AF mouthwash gave 28%–30% reductions. The placebo and competitive formulas were all below 25% reductions in measurable LPS (Figure 5b).

### 3.6 Twelve‐hour bacterial regrowth kinetics

The regrowth study showed that relative to the untreated biofilms and biofilms treated with the negative control, the regrowth of bacteria derived from the AZF‐treated biofilms was significantly reduced or inhibited. This is based on the reduced bacterial culture absorbance over the 12‐h time period as measured per hour (Figure 5). Relative to the untreated and negative control‐treated biofilms, AZF treatment of biofilms significantly inhibited bacterial regrowth (*p* < .001) beginning at 2 h and continuing for 12 h. No observed difference in antibacterial activity was documented for the negative control mouthwash group when compared with the untreated biofilm group at all time points.

### 3.7 Zinc deposition on MatTek EpiGingival^TM^ tissues

While the SIKT results suggest that the immediate antimicrobial effects of the tested formula can be traced largely to the presence of AmF, it is known that in other formulas, Zn is readily deposited on oral soft tissues and provides a reservoir of longer lasting activity against bacteria. In order for this to be a relevant mode of action in this formula, a significant amount of Zn ions must be able to be deposited on oral soft tissue substrates. As can be seen in Figure 7, approximately 1.1 ㎍/cm^2^ of Zn ions was detected on MatTek model gingival tissues treated with the test mouthwash. No background Zn deposition was observed from the control formulas.

### 3.8 Zinc deposition imaging

While in vitro deposition of Zn is important, this does not always translate to in vitro deposition of meaningful levels of Zn retention. To confirm that this effect could also be detected on real gum tissue, superficial cells were harvested by swabbing the gums of human volunteers. Cells treated with mouthwash for 1 min were then stained with a Zn‐sensitive dye. Images of these cells can be seen in Figure 8. AZF‐treated human cells stained significantly with the Zn indicator stain while background levels of Zn were not detectable on those cells treated with the AF mouthwash.

## 4 DISCUSSION

Plaque‐induced gingivitis is a very common disease, with recent estimates suggesting that it affects more than 75% of the population (Ababneh et al., 2012; Carvajal et al., 2016; Elias‐Boneta et al., 2018; Li et al., 2010). Mechanical plaque control in conjunction with an antibacterial toothpaste is often used to help control plaque and gingivitis (Delgado et al., 2018; Hu et al., 2019). Regular use of antibacterial mouthwashes acts as a supplement to reductions in plaque beyond that seen by tooth brushing alone. While many mouthwash formulas are able to provide immediate, direct antibacterial action, it is desirable to provide both antibacterial and host tissue benefits from a single rinse formula. The current formulation provides a balance of both host and bacterial benefits, leading to improved plaque and gingivitis efficacy (Montesani et al., 2024).

Salivary SIKT experiments provide a relatively simple means of screening formulations for broad‐spectrum antibacterial activity. While the culturable fraction of the oral microbiome is not considered complete, it is representative of a diverse selection of the organisms present in the oral cavity. Combined with the easiest accessibility, therefore, it is a convenient method to evaluate how a mouthwash works against real‐world bacterial isolates, as opposed to the more readily susceptible organisms generally found in laboratory cultures. As shown in Figure 1, the test mouthwashes performed significantly better than a matched placebo mouthwash with the majority of the short‐term effect driven by the amine. To further probe this immediate antibacterial activity, a fluorescent method that capitalizes on the permeabilization of cell membranes was also used (Figure 2). Traditional SIKT methods rely on diluting and plating of bacteria; however, dilution can allow highly soluble ingredients to be extracted from the cell membrane and the bacteria can recover, thereby not reflecting the actual antibacterial potential of the active system. By using the BacLight LiveDead two‐component staining system, antibacterial efficacy can be measured without diluting and plating. In this technique, bacteria are stained with propidium iodide, a membrane‐impermeant, fluorescent, DNA‐intercalating dye, and then counterstained with SYTO9, a green fluorescent DNA dye that can penetrate cell membranes. Cells with damaged cell membranes will be stained red while all cells will stain green. The relative fluorescence of the two dyes can then be used to quantify the antibacterial effect in the bacterial community. Unlike plating, this staining method works with equal efficiency for the majority of the species of bacteria in a mixed population, allowing for enumeration of a more diverse community. A representative community of five species of bacteria commonly found in the oral cavity representing Gram negative and Gram positive, as well as anaerobic and aerobic species, was chosen for this research. Additionally, we have early, middle and late colonizing members of the biofilm community, giving broad representation of the oral community. In this experiment, the AZF mouthwash, performed similarly to the positive control, 100% EtOH, and significantly better than the placebo mouthwash (Figure 2). Additionally, the AF mouthwash performed similarly to the AZF formula in this assay (Figure 2), which reinforces the finding that the immediate efficacy of this formula is driven largely by the amine while ZnL drives long‐term effects and that the killing of bacteria by the AmF mouthwash occurs via a permeabilization of bacterial cell membranes.

While the immediate efficacy of this formula is clearly driven by the presence of the amine, ZnL has been included in the new formula to provide a long‐term effect, in addition to the well‐known soft tissue benefits of zinc. One well known effect of zinc compounds is the inhibition of bacterial metabolism. The in vitro plaque glycolysis assay (Figure 3) probes the ability of formulas to interrupt the metabolic activity of bacterial communities, using acid production as an easy measure of metabolism. As can be seen in Figure 3, the AZF mouthwash inhibited the metabolic activity of saliva‐derived biofilms following a single treatment and was statistically significantly different (*p* < .05) from the placebo and PBS by Student's *t* test.

In the aerobic biofilm model (Figure 4), consumer usage conditions are mimicked by treating the biofilm two times per day for 30 s per treatment. This method allowed the benefits of increased exposure to be seen as well as helping to understand how the test ingredients impact highly structured, complex communities. The AZF mouthwash provided greater effect than either the AF mouthwash or the ZnL mouthwash.

In addition to the traditional antimicrobial activities of mouthwashes that are associated with plaque and gingivitis benefits, the inclusion of ZnL in this formula provides an opportunity to go directly after one of the key promoters of oral tissue inflammation, LPS. LPS is known to be a strong stimulator of oral inflammatory responses and, ultimately, gingivitis, (Sismey‐Durrant & Hopps, 1991), and Zn ions are known in the literature as potential neutralizers of LPS (Stanton & McCracken, 2021).

Using a commercially available LPS quantification method, we were able to demonstrate that the formulated mouthwash was able to significantly neutralize *E. coli* LPS (Figure 5a) as well as the more clinically relevant LPS from *P. gingivalis* (Figure 5b). In both cases, the AZF mouthwash was able to significantly reduce the amount of measurable LPS. This effect was not seen with an AmF‐alone control, suggesting that the LPS neutralization is driven by the presence of ZnL in this formula. Competitive mouthwashes containing chlorhexidine digluconate or essential oils were also unable to provide this benefit.

As LPS is known to stimulate the early inflammation associated with gingivitis, we can surmise that the ability to neutralize LPS will contribute to the prevention of gingivitis potentially caused by any remaining bacteria and bacterial by‐products. Taken together, these data suggest that the tested mouthrinse goes beyond typical antibacterial formulations to provide additional gingivitis prevention through the neutralization of one of the major drivers of gingival inflammation in the oral cavity.

**Figure 6:**
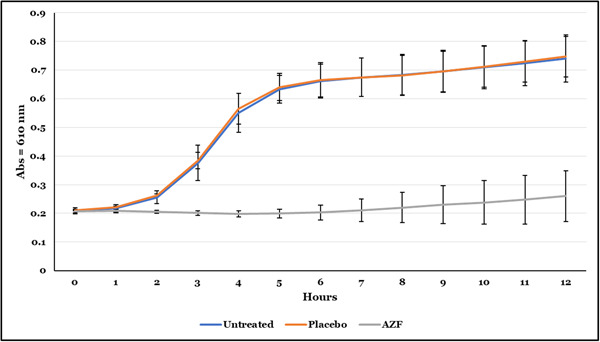
Comparison of 12‐h bacteria regrowth posttreatment of biofilms with AZF mouthwash to biofilms treated with placebo mouthwash and to the untreated biofilm. Each data point is an average over three experiments with 4 data points obtained per experiment. Bacterial growth was measured using optical density (OD) via absorbance at 610 nm. Bacterial growth on biofilms treated with the AZF mouthwash was statistically significantly (*p* < .0001), lower than either the bacterial growth on the untreated biofilm or on the placebo‐treated biofilm starting at 2 h and continuing for all later time points.

Finally, the longevity of antibacterial effectiveness of the mouthwashes were evaluated in a 12‐h antibacterial regrowth model (Figure 6). The AZF mouthwash was effective at inhibiting bacterial regrowth for up to 12 h. The short‐term and long‐term data presented above demonstrate that the combination of an amine and ZnL provides considerable antibacterial efficacy in a mouthwash formulation over an extended time.

**Figure 7:**
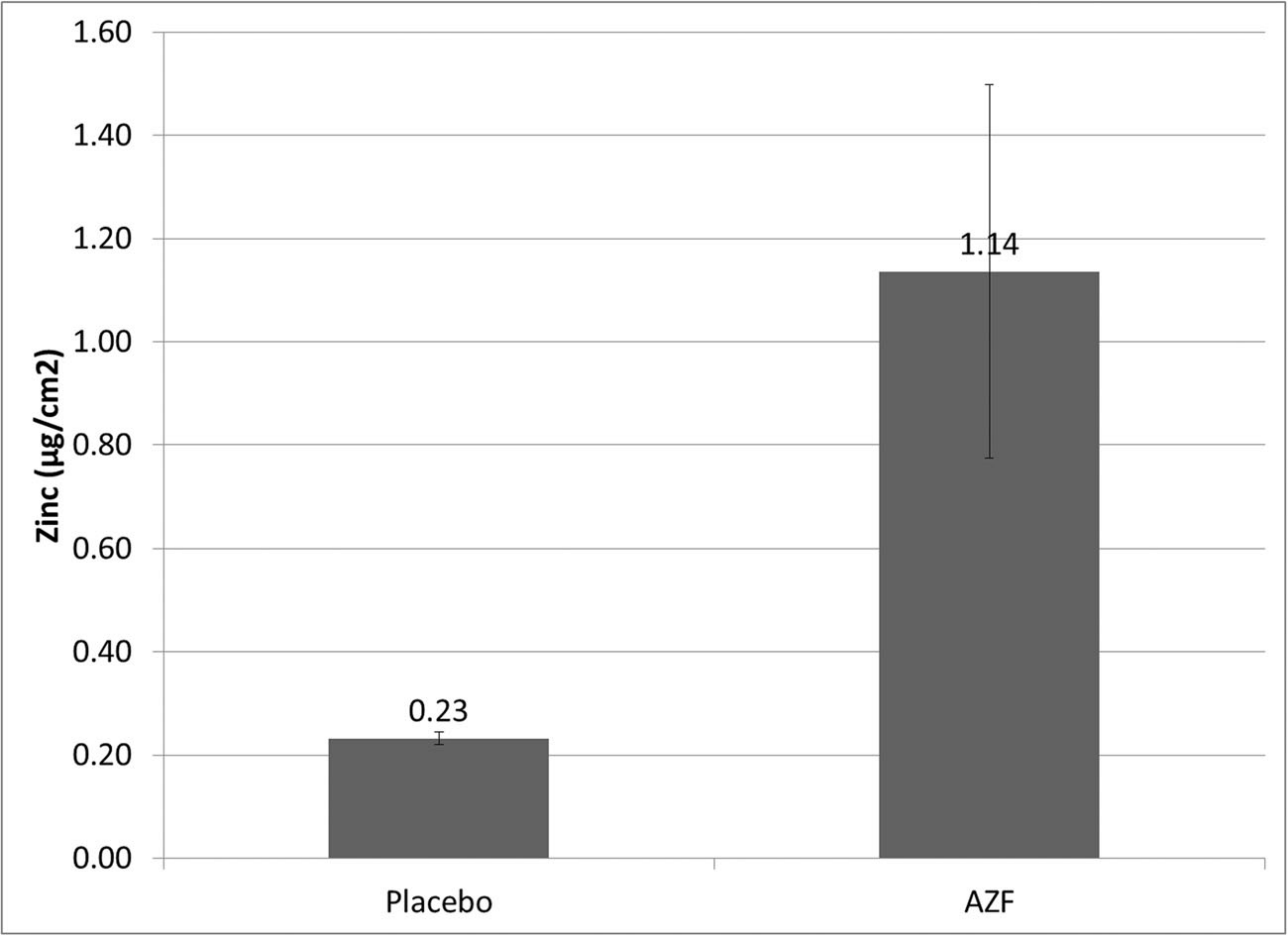
Uptake and retention of Zn ions on mouthwash‐treated tissues. Model gingival tissues were treated with mouthwash for 2 min at room temperature, washed, and then total Zn in tissues was measured. The AZF mouthwash is able to deliver about 1.1 ㎍/cm^2^ of Zn to model tissues. This suggests that the Zn from the mouthwash is delivered not only to bacteria but to the oral soft tissues, which can serve as a reservoir of Zn ions.

**Figure 8:**
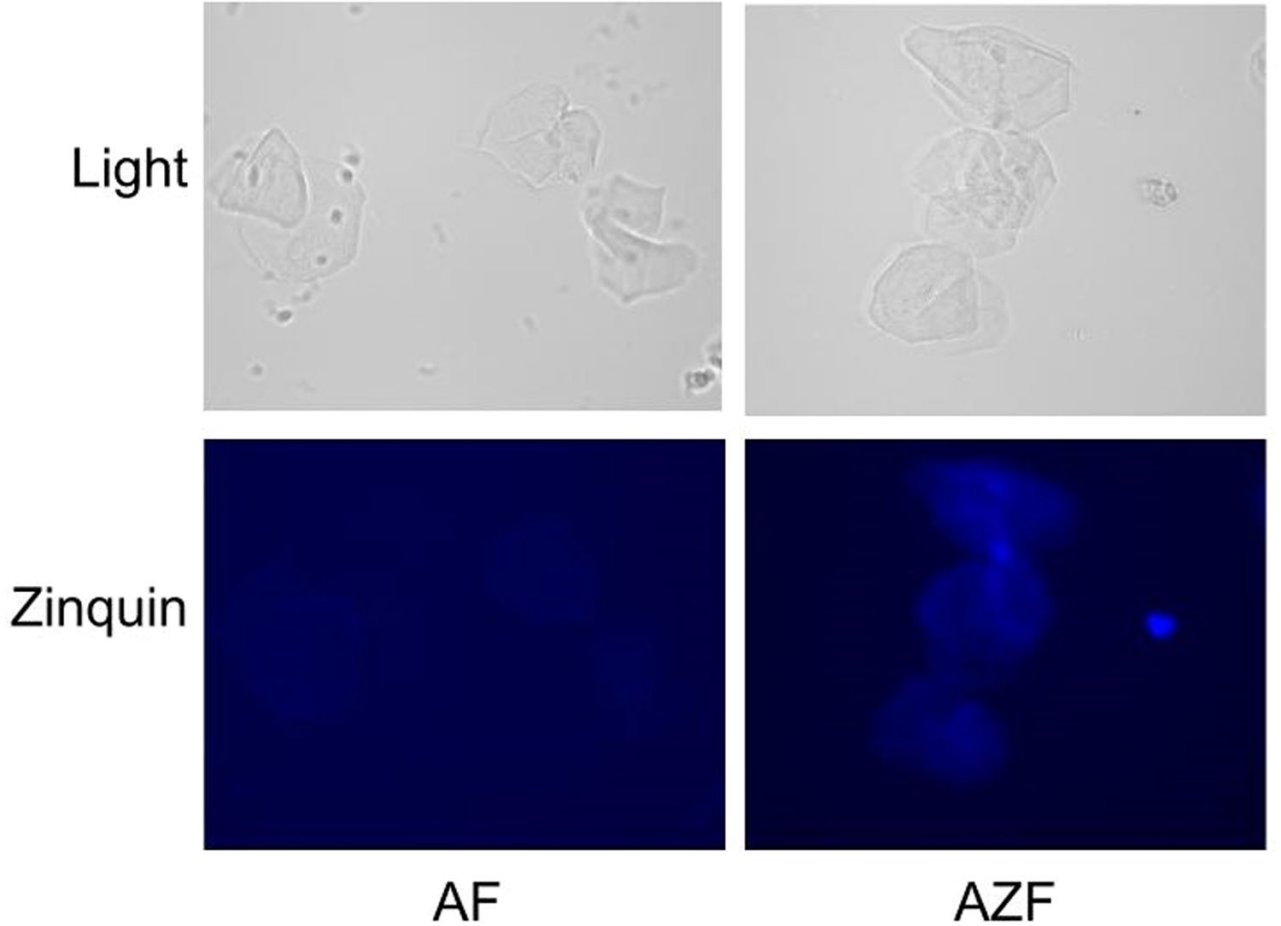
Zn deposition on cheek cells following mouthwash use. To confirm that the observed Zn deposition was able to occur under real use conditions, volunteers rinsed with AZF mouthwash or a control AmF‐only mouthwash. Cheek cells were then harvested and stained to visualize Zn. Patients who rinse with the AZF mouthwash had observable Zn ions detected on harvested cheek cells.

One of the most valuable aspects of zinc as an oral care agent is its ability to be taken up on oral soft tissues, which can serve as a reservoir for longer lasting efficacy. We used the MatTek EpiGingival^TM^ tissue as a model for oral soft tissues to understand if the ZnL in this formula was available in a form that could be taken up by oral soft tissues. As demonstrated in Figure 7, about 1.1 ㎍/cm^2^ of Zn ions was retained on the surface of treated tissues. Additionally, we were able to visually demonstrate this Zn delivery to oral soft tissues following actual usage of the formula by volunteers (Figure 8). Taken together, these data suggest that the oral soft tissues will be able to provide a reservoir of Zn for longer lasting effects.

We used a variety of methods to create a comprehensive picture of how this formula might function within the diverse niches of the oral cavity. The combination of simple and complex models allows us to understand both the mechanism and degree of efficacy. By looking at a mixture of both aerobic and anaerobic biofilms, we created we demonstrate the impacts of this formula on the broad spectrum of bacterial species found in the oral cavity, in their most protected state. The results presented here illustrate the in vitro antimicrobial and soft tissue benefits of a novel mouthwash formulation containing amine fluoride and ZnL. Overall, we have demonstrated that this formula acts both through the immediate killing of bacteria, as demonstrated in the SIKT tests, and through longer lasting effects, as seen in the biofilm studies. Additionally, the retention of Zn on oral soft tissue surfaces suggests an ability to provide a long‐term reservoir of Zn for delayed release effects. Finally, the addition of ZnL to this formula provides an additional protection against gingivitis by neutralizing the highly stimulatory LPS of Gram‐negative bacteria. This formula is a clear candidate for boosting oral health through an extended dental hygiene routine coupled with daily flossing. Confirming this, this formula was shown to provide significant reductions in both plaque and gingivitis measures in a 6‐month randomized controlled clinical trial (Montesani et al., 2024).

## AUTHOR CONTRIBUTIONS

**Lyndsay M. Schaeffer**: Conceptualization; methodology; investigation; formal analysis; writing. **Ying Yang**: Methodology; investigation; formal analysis; visualization. **Carlo Daep**: Methodology; investigation; writing—review and editing. **Ekta Makwana**: Methodology, investigation; formal analysis. **Golnaz Isapour**: Resources; investigation. **Norbert Huber**: Resources; supervision; project administration.

## CONFLICT OF INTEREST STATEMENT

All authors are employees of the Colgate‐Palmolive, Co., which funded this research.

## Data Availability

Research data are not shared.
